# Isolation, characterization, and optimization of extracellular laccase-producing bacteria from dye-contaminated textile wastewater for dye decolonization

**DOI:** 10.1371/journal.pone.0354516

**Published:** 2026-09-29

**Authors:** Ali Seid, Fisseha Getachew, Marye Alemu, Birhanu Andualem

**Affiliations:** 1 Collage Natural and Computational Science Department of Biotechnology, Woldia, Ethiopia; 2 Department of Biotechnology, Institute of Biotechnology, University of Gondar, Gondar, Ethiopia; Government College University Faisalabad, PAKISTAN

## Abstract

Textile industries are important in both the global economy and our daily lives. They are, however, simultaneously becoming one of the main sources of environmental pollution in the world. It needs a greater amount of water for wet textile operations such as dyeing, and as a result, produces significant dosages of wastewater. Textile wastewater is highly colorful and contains dyes that are highly harmful to living creatures, and is discharged to the environment every day. The objective of the present study was to isolate and characterize extracellular laccase-producing bacteria from textile dye-contaminated wastewater to decolorize the dye. Samples were collected from the dye-contaminated wastewater sample site, including dye centers (DC), dye printing (DP), dye mixers (DM), and dye drains (DD). To isolate extracellular laccase producing bacteria from dye contaminated wastewater sample, 1 ml of sample was taken and serially diluted in physiological saline solution. Then, 0.1 ml from each diluted test tube was spread over nutrient agar and incubated. Then, the colonies were sub-cultured. Ten bacterial isolates were identify and designated as DMC1, DMC2, DMC3, DMG3, DMW1, DMY, DDY, DPG, DPC, and DCC. These bacterial isolates were tested for laccase production, and only four of them (DMC2, DMC3, DMG3, and DMW1) gave a positive result. Using morphological and biochemical characters, the four isolate were identified as a presumptive Bacillus species. Laccase production by isolates was optimized, and Maximum laccase production and dye decolonization were achieved at pH 7, a temperature of 42°C, and a dye concentration of 0.05 g per 50 mL of culture mediumTherefore, the enzyme produced, using the potential strain is capable of decolorizing textile dyes (black, yellow, and red). As a result, crude laccase enzyme from these bacterial isolates could be employed since a viable alternative to the physical and chemical processes of textile dye, as they have a high potential for decolorizing the dyes in this study. Scale up the production of laccase enzyme at certain industrial level is significant. Laccase enzyme should be purified whenever possible. Furthermore, molecular identification can be used to correctly identify up to strain level. The information gathered in this study could be used as a baseline for treating dye-polluted and contaminated environments.

## 1. Introduction

The textile industry plays a crucial role in the global economy by contributing significantly to economic growth, employment generation, and social development. It has helped lift millions of people out of poverty worldwide through large-scale job creation and industrial expansion [1]. Despite these socioeconomic benefits, the textile sector is recognized as one of the major contributors to environmental pollution, particularly water pollution. The industry is among the largest consumers of freshwater resources, and the increasing global demand for textile products has led to a corresponding rise in textile wastewater generation, making textile effluents one of the most serious environmental challenges globally [2]

A major source of pollution from textile industries arises during the dyeing and finishing processes, where substantial quantities of dyes are discharged into wastewater. It is estimated that a significant proportion of dyes used in textile processing are not fixed onto fabrics and are released into effluents, resulting in highly colored wastewater [3]. Historically, dyes were derived from natural sources such as plants, insects, and minerals. However, due to increased industrial demands, synthetic dyes derived from petrochemicals have largely replaced natural dyes. Synthetic dyes are preferred because of their high solubility in water, rapid color fixation, wide color range, and cost-effectiveness [4]. Unfortunately, these dyes are chemically stable, resistant to degradation, and often toxic, making their removal from wastewater difficult.

The discharge of untreated or inadequately treated colored textile wastewater has severe impacts on aquatic ecosystems and surrounding environments. Textile effluents reduce light penetration in water bodies, disrupt photosynthetic activity, alter physicochemical properties of water, and pose serious threats to aquatic organisms, agriculture, aquaculture, and public health [5]. Consequently, colored wastewater pollution has become a global environmental concern, emphasizing the urgent need for efficient, sustainable, and eco-friendly wastewater treatment technologies.

In Ethiopia, the textile industry is one of the fastest-growing industrial sectors and plays a significant role in national economic development. Favorable climatic conditions for cotton production, availability of raw materials, and access to low-cost labor have accelerated the expansion of textile manufacturing in the country. While industrial development contributes to economic growth and employment opportunities [6], environmental pollution associated with textile wastewater has emerged as a critical challenge. Many textile factories in Ethiopia discharge large volumes of dye-containing effluents directly into public drains or nearby open environments without adequate treatment [3]. The water consumption and wastewater generation from textile industries depend largely on the processing operations used during dyeing and finishing stages, during which large quantities of dyes are released into wastewater [7]

Conventional physicochemical methods, including coagulation–flocculation, adsorption, oxidation, and membrane filtration, have been widely applied for dye removal from textile wastewater [8]. Although these methods can achieve partial decolorization, they suffer from several limitations such as high operational costs, excessive chemical consumption, incomplete dye removal, generation of secondary hazardous sludge, and reduced efficiency for certain dye classes like reactive, acid, and direct dyes [9] These drawbacks highlight the need for alternative treatment strategies that are both environmentally friendly and economically viable.

Biological approaches, particularly enzyme-mediated dye degradation, have emerged as promising alternatives to conventional methods. The use of microorganisms and their enzymes for textile dye decolorization offers several advantages, including low cost, minimal chemical input, reduced sludge formation, and production of non-toxic end products [10]. Among various enzymes, laccases (EC 1.10.3.2) have attracted considerable scientific attention due to their strong oxidative capability and broad substrate specificity. Laccases are multicopper oxidases that catalyze the oxidation of a wide range of phenolic and non-phenolic compounds using molecular oxygen, leading to the degradation and detoxification of synthetic dyes [11]. Dye-degrading enzymes play a crucial role in the bioremediation of textile and industrial dye pollutants. Among the most important are lignin peroxidase (LiP), manganese peroxidase (MnP), azoreductase, tyrosinase, and versatile peroxidase (VP). LiP and MnP are extracellular oxidative enzymes capable of degrading complex aromatic dye structures, while VP combines the catalytic properties of both enzymes, enhancing substrate versatility. Azoreductase specifically cleaves azo bonds, the major chromophoric groups in azo dyes, leading to decolorization. Tyrosinase catalyzes the oxidation of phenolic compounds, contributing to dye transformation and detoxification. Together, these enzymes offer eco-friendly solutions for wastewater treatment.Bacterial laccases are particularly advantageous for industrial and environmental applications because they exhibit higher stability under extreme conditions such as alkaline pH, high temperatures, and the presence of halides compared to fungal laccases [12]. These properties make bacterial laccases highly suitable for textile wastewater treatment and bioremediation processes. Consequently, isolating and characterizing efficient laccase-producing bacteria from dye-contaminated environments represents a promising approach for sustainable textile dye decolorization [13].Therefore, this study was to isolate, screen and characterize laccase-producing bacteria from textile dye-contaminated wastewater Textile Factory for the purpose of decolorizing Textile dye

## 2. Materials and methods

### 2.1. Description of the study area

The study was conducted at Cellular and Microbial Laboratory, Institute of Biotechnology, University of Gondar. Gondar is situated in Amhara region, far from 738 km from Addis Ababa, the capital city of Ethiopia.

### 2.2. Study design

Experimental study was conducted for isolation and characterization of laccase producing bacterial isolates.

### 2.3. Sample collection and processing

Purposive sampling technique was employed for the collection of textile dye contaminated wastewater sample from dye center (DC), dye printing (DP), dye mixer (DM), and dye drain (DD) in Bahir Dar Textile Factory. A total of four samples from each sampling sites were collected in sterile glass bottles, each containing 100 ml of sample and transported in ice box to University of Gondar, Institute of Biotechnology; Cellular and Microbial Laboratory and stored at 4∘C until used.

#### 2.3.1 Reagents and chemicals.

The reagents and chemicals used in the study included nutrient agar components (peptone, beef extract, sodium chloride, and agar), physiological saline water, tannic acid, glucose, yeast extract, calcium carbonate (CaCO_3_), copper sulfate (CuSO_4_), magnesium sulfate (MgSO_4_), ferrous sulfate (FeSO_4_), zinc sulfate (ZnSO_4_), and textile dyes (yellow, black, and red). Various biochemical test reagents were also employed, including hydrogen peroxide (3% H_2_O_2_), urea agar, Triple Sugar Iron (TSI) agar, MR-VP broth, methyl red indicator, α-naphthol, potassium hydroxide (KOH), Simmons citrate agar, tryptophan broth, and Kovac’s reagent. Additional media components used manuscriptfor laccase production included tryptone and buffer solutions for pH adjustment. These chemicals and reagents were essential for bacterial isolation, screening, biochemical characterization, optimization of laccase production, and evaluation of dye degradation potential

### 2.4. Isolation of laccase secreting bacteria

One ml of dye-contaminated wastewater samples were taken and serially diluted in each test tubes, physiological saline water was used as diluents., in the order of 10^−1^ −10^−7^. Next this, 0.1 ml from each diluted test tube was spread over nutrient agar (Peptone 5 g/L, Beaf extract 3 g/L, NaCl 5 g/L, agar 15 g/L) [14]. Then, inoculated plates were incubated for 48hrs at 37∘C. Following incubation, different bacterial colonies developed and were identified based on colony shape, color and size. To get pure cultures, colonies were streaked using the streak plate method [15]. There were ten distinct pure bacterial isolates found. The isolates were then transferred to nutrient agar with 0.5 percent tannic acid for preliminary screening.

### 2.5. Primary screening of isolates

The isolates were primarily screened for laccase production by inoculating them on nutrient agar plate (Peptone 5 g/L, Beaf extract 3 g/L, NaCl 5 g/L, agar 15 g/L) containing 0.5% tannic acid medium. The inoculated plates were incubated at 37∘C for 48hrs. After incubation, the existence of a black color zone encircling the bacterial growth verified laccase production, and all ten isolates with a black color zone were chosen and subjected to secondary screening [16]

### 2.6. Secondary screening of isolates in mineral salt medium (MSM) Agar medium

The bacterial isolates were further screened to select the best isolates produced laccase by using mineral salt medium (MSM) solid medium containing glucose 0.1 g/L, yeast Extract 0.2 g/L, NaCl 0.1 g/L, CaCO_3_ 0.02 g/L, CuSO_4_ 0.002 g/L, MgSO_4_ 0.002 g/L, FeSO_4_ 0.001 g/L and ZnSO_4_ 0.009 g/L supplemented with 0.1 g/L of each dye (yellow, black and red) as a source of hydrocarbon and then incubated at 37∘C for 48hrs. Laccase synthesis by isolates was confirmed after incubation by the appearance of a distinct zone of dye hydrolysis around the bacterial growth. This is an indicator of dye degradation and laccase secretion. The diameters of each clear zone were measured three times in different directions using a ruler in mm, and the average was taken [17]. The larger the clear zone (diameter) around bacterium growth is the more laccase generated, and the bacterium is chosen for future research.

### 2.7. Characterization and identification of the selected isolates

**Morphological Characterization:** The isolates were morphologically classified based on their macroscopic appearance (i.e., Colonial morphology such as size, color, and shape) were investigated [18]. Bergey's Manual of Systematic Bacteriology was used to identify the most laccase-producing bacteria based on visual and biochemical traits.

**Motility Test: Fresh:** 24hrs old bacterial colony was inoculated into motility test agar medium using the stab method vertically top to bottom through the middle of test tubes, then incubated for 24hrs at 37 ∘C. Highly motile bacteria species growth was spread throughout the tube after 24 hrs, whereas non-motile strains’ development was detected solely along the stab line [19]

### 2.8. Biochemical identification of isolates

A loop of an overnight culture was put on nutrient agar plate and incubated for 24hrs at 37∘C. TSI, urease, citrate utilization, oxidase, catalase, H_2_S gas generation, indole production, methyl red, and VP (Vogues Proskauer) were among the assays carried out [20]. All biochemical experiments were carried out according to the instructions in Bergey's manual of determinative bacteriology. Each test was briefly explained below separately.

**Oxidase test:** Loop was used to inoculate bacterial cultures onto oxidase disc. A positive test was defined as the production of blue or purple color after 30 seconds.

**Catalase test:** A few drops of hydrogen peroxide (3%) were applied to the surface of a slide containing bacterial culture, and the effervescence was detected within 5–10 seconds, which was interpreted as a positive reaction induced by the liberation of free oxygen as gas bubbles. Catalase production was measured by inoculating a 24hrs starch agar slant culture with 1–3 ml of 3 percent H_2_O_2_ and using an un-inoculated medium as a negative control. The presence of bubbles signifies a positive catalase test, whereas the lack of bubbles indicates a negative test.

**Urease production test:** When urea is hydrolyzed, ammonia accumulates in the medium and makes it alkaline. This increase in pH causes the indicator to change from orange-red to deep pink or purplish red (cerise) and is a positive test for urea hydrolysis. Failure of a deep pink color to develop is a negative test [21]. Inoculated urea agar slants were inoculated with bacterial isolates and incubated for 48hrs at 37∘C. The transformation of hue from yellow to red or deep pink was interpreted as a favorable reaction for urea breakdown via the creation of the enzyme urease.

**Triple Sugar Iron Agar Test (TSI):** Triple sugar iron agar slants were inoculated with bacterial isolates and incubated at optimum temperature about 48hrs. Change in color of both and slants to pink and butt to yellow was taken as positive to ferment glucose and not to ferment sucrose or lactose whereas if both slant and butt were pink in color means none of the sugar was fermented, both butt and slant change to yellow was taken as positive to ferment glucose and sucrose or lactose with bubbles and H_2_S gas formation was checked.

**Methyl red and Voges-Proskauer (MR-VP) test**

I. MR-VP broth (1% glucose supplemented in nutrient broth).
II. Methyl red (0.1 g methyl red dissolved in 300 ml of 95% ethanol).
III. VP reagent I (6 g α-napthol dissolved in 100 ml of 95% ethanol)
IV. VP reagent II (40% KOH)

The tubes containing MR-VP broth (1% glucose supplemented in nutrient broth I) were inoculated with bacterial isolate and incubated at optimum temperature for 12hrs and 24hrs. Five drops of methyl red (0.1 g methyl red dissolved in 300 ml of 95% ethanol II) were added to one of the tubes and change in color (brick red) if any was recorded. To the other tube, 0.6 ml of VP reagent I and 0.2 ml of VP (6 g α-napthol dissolved in 100 ml of 95% ethanol were added. After shaking the tubes gently with their caps off to expose them to air, these were allowed to stand for 30mins and the change in the color from pink to red was taken as positive and no change in color or black color formation was taken as negative.

**Methyl Red (MR) Test:** The methyl-red tests are used to determine whether or not a bacteria can do mixed-acid fermentation. Glucose, peptone, and a phosphate buffer are all included in MR-VP broth. When organisms undergo mixed-acid fermentation, they create enough acid to overwhelm the broth's buffering capability, resulting in a pH drop. Other types of fermentation organisms are unable to overcome the broth's buffering capability. In the test Tubes, isolates were inoculated into 10 ml of sterilized MR-VP broth and incubated for 24 hours at 37∘C. In each inoculated and un-inoculated tube, 5 drops of Methyl Red indicator were applied. A positive test was indicated by the presence of a brilliant red hue.

**Voges-Proskauer Test:** It is a test used to detect acetone in a bacterial broth culture. The test depends on the digestion of glucose to acetyl methyl carbinol. If glucose is being broken down, it will react with alpha-naphthol (VP reagent #1) and potassium hydroxide (VP reagent #2) to form a red color. Tubes with 10 ml of MR-VP broth were inoculated with each isolates. After incubation, 5 drops of naphthol and potassium hydroxide reagent was added to the tubes. The mixture was shaken vigorously for few minutes and allowed to stand for two hours and positive reaction was given by the appearance of deep rose color.

**Citrate utilization test:** Simmon's citrate agar slants were inoculated with bacterial isolates and incubated at optimal temperature for 48hrs, with a change in the medium's color from green to blue being considered a positive test. The citrate test is used to distinguish between intestinal bacteria based on their capacity to utilize carbon dioxide as their only carbon source. Citrate utilization is dependent on the presence of the enzyme “Citrate Permease,” which is created by the organism and aids in the transfer of citrate into the cell. Prepare Observe the tubes for color change from green to blue [22].

**Indole production test:** Tryptophan is an essential amino acid that is oxidized by certain bacteria, resulting in indole, pyrivic acid, and ammonia production. The organisms were inoculated and cultured in 10 ml of sterilized Tryptophan broth distributed in test tubes for 24hrs at 37∘C. After the tubes have been incubated, add 1 ml of Kovac's reagent to each one. A good response was shown by the emergence of a cherry red color ring in the surface layer [23]

### 2.9. Optimization of parameters for laccase production

Optimization of pH, temperature and substrate concentration is useful in production of maximum laccase enzyme and to determine the optimum level of each taken in the study. It is also less time consuming, reduces the cost and improve the product yield [24]. Laccase production media (LPM) g/L containing Tryptone 0.02, Yeast Extract 0.01, Sodium Chloride 0.01, CaCO_3_ 0.02, MgSO_4_ 0.002, FeSO_4_ 0.01 and ZnSO_4_ 0.009 supplemented with concentration of dye (yellow, black and red) were prepared in experimental flasks [25].

#### 2.9.1 Effect of temperature.

After autoclaved, the bacterial inoculum with similar turbidity of McFarland standard 3 (9 ×10^8^ FCA) was added in 50 ml of laccase production medium (g/L) and incubated at four different temperatures, namely 32∘C, 37∘C, 42∘C and 47 ∘C at 150 rpm using orbital shaking incubator. After four days of incubation, fermented broth were collected and centrifuged at 10,000 for 10mins, and then the cell free culture supernatant was collected as crude enzyme extracts and the laccase concentration were determined using spectrophotometer at 280nm against the dye meal media as a blank. The temperature at which high laccase concentration observed was selected as optimum temperature for the corresponding bacterial isolates.

#### 2.9.2 Effect of pH.

The optimum pH for the growth and laccase production of isolates were studied by adjusting the initial pH of the laccase production medium (g/L), such as 5, 6, 7 and 8 using buffer and the flasks containing 50 ml of each laccase producing broth media were autoclaved at 121∘C for 15mins. The bacterial inoculum with similar turbidity of McFarland standard 3 (9 ×10^8^ FCA) was added in 50 ml of the laccase production medium and cultured on a rotary shaking incubator with 150 rpm for 96hrs. After four days growth, the fermented broth were collected and centrifuged at 10,000 for 10mins, and then the cell free culture supernatant was collected as crude enzyme extracts and the laccase concentration was determined using spectrophotometer at 280nm against the dye meal media as a blank. The pH at which high laccase concentration observed was selected as optimum pH for the corresponding bacterial isolates.

#### 2.9.3 Effect of dye concentrations.

The best concentration of dyes (black, yellow and red) for maximum laccase production were determined by using laccase producing media (g/L) supplemented with different concentrations of dye (0.05 g, 0.1 g, and 0.15 g) were prepared in experimental flasks containing 50 ml of laccase producing broth media. The bacterial inoculums with similar turbidity of McFarland standard 3 (9 ×10^8^ FCA) was added in 50 ml of medium and incubated at 150 rpm using orbital shaking incubator. After four days growth the fermented broth were collected and centrifuged at 10,000 for 10mins, and then the cell free culture supernatant was collected as crude enzyme extracts and the laccase concentration were determined using spectrophotometer at 280nm against the dye meal media as a blank. The substrate concentration which has better laccase concentration was taken as optimum substrate.

### 2.10. Percentage dye decolorization assay by crude laccase enzyme extract

Crude laccase enzyme extracts were investigated for dye decolorization of selected three individual dyes namely black, yellow and red. Then, 0.05 gm of each dye was solubilized in 25 ml of distilled water in experimental flask. The reaction mixture was 25 ml of individual dye solution and 25 ml of crude laccase enzyme extract/ enzyme followed by incubation at 42∘C for 48hrs. Control samples were run in parallel without addition of crude laccase enzyme. The decolorization percentage was determined spectrophotometrically as the relative decrease of absorbance at each maximal absorbance wavelength of the dyes. The crude laccase enzyme was used as blank. All reactions were performed in triplicates. Dye decolorization was expressed in terms of percentage calculated according to the equation [26].


$$\begin{array}{c} \%\text{Decolorization}=\frac{𝐀𝐎-𝐀𝐭}{AO}\times 100 \end{array}$$

Where, A_0_ is an absorbance at an appropriate wavelength of the dye immediately measured after adding the enzyme solution and A_t_ is an absorbance after incubation.

### 2.11. Data analysis

The experimental activity was conducted in triplicates under aseptic condition. The data were analyzed using the SPSS software package version 22. Means and standard deviations of the triplicate data analysis were calculated via one-way analysis of variance (ANOVA) at 95% of significance level with P value of <0.05 was considered as statistical significance.

## 3. Results

### 3.1. Isolation and identification of laccase producing bacteria

In the present study, ten bacterial isolates were isolated and designated as DMC1, DMC2, DMC3, DMG3, DMW1, DMY, DDY, DPG, DPC, and DCC. Isolate DMC1, DMC2, DMC3, DMW1, DMY2 and DMG3 were isolated from dye mixer, and DDY and DCC were isolated from dye drain and dye center samples respectively. The remaining two isolates, DPC and DPG were isolated from dye printing sample. Then all isolates were screened for laccase production and the result is presented below in the text.

### 3.2. Primary screening of isolates for laccase production

All isolates were screened for laccase production in nutrient agar containing 0.5% tannic acid and incubated at 37∘C for 48hrs. All isolates were showed black colors zone, surrounding the bacterial growth as indicated in Fig 1. Thus, all isolates were chosen for secondary screening.

**Fig 1:**
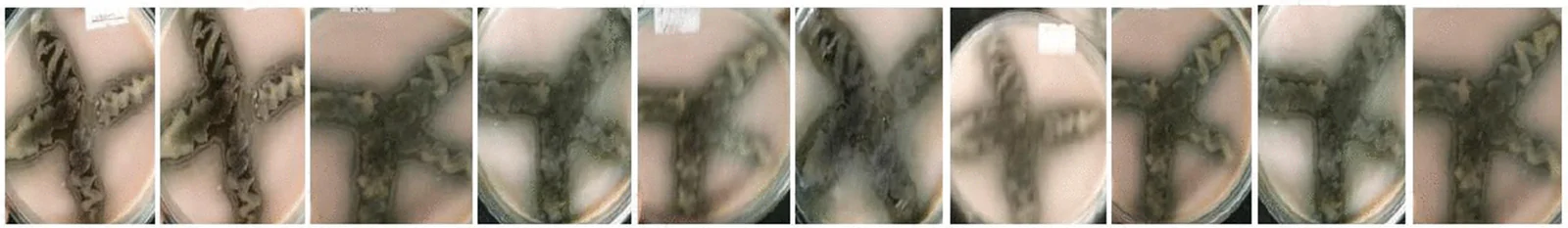
Oxidation of tannic acid by ten isolates.

### 3.3. Secondary screening of isolates

Ten bacterial isolates were further screened on mineral salt medium (MSM) containing (g/L) glucose 0.1, yeast extract 0.2, NaCl 0.1, CaCO_3_ 0.02, CuSO_4_ 0.002, MgSO_4_ 0.002, FeSO_4_ 0.01 and ZnSO_4_ 0.009 supplemented with 0.1 g of each dye (yellow, black and red) as a source of hydrocarbon and then incubated at 37∘C for 48hrs. After 48hrs of incubation, four isolates (DMC2, DMC3, DMG3, and DMW1) showed a distinct clear zone of the dyes with diameters of 14.3 mm, 13.3 mm, 9.3 mm, and 12.3 mm, respectively. This demonstrates that these isolates can breakdown the dye, which indicates laccase enzyme production as indicated in Fig 2.

**Fig 2:**
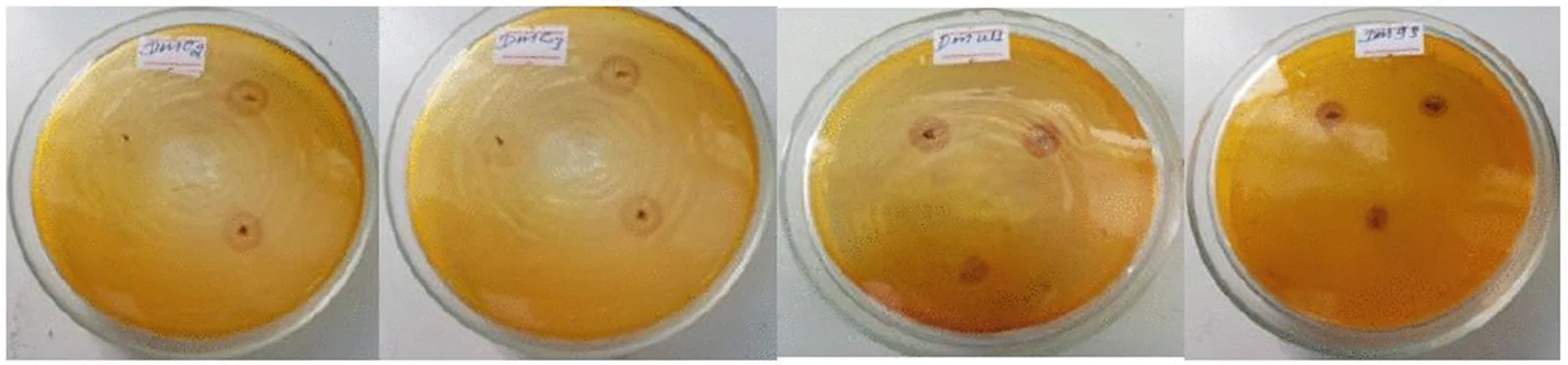
Clear zones around bacterial isolates growth.

### 3.4. Morphological characterization of selected isolates

#### 3.4.1. Colony morphology of isolates.

Cultural characterization of isolates revealed that all isolates were circular in shape with translucent texture. Indeed DMC2 is large, raised, mucouid and creamy, whereas DMC3 is small, creamy and raised. Of the rest isolates, DMW1is white, flat, and translucent and isolate and DMG3 is large and grey.

#### 3.4.2. Microscopic characteristics of isolates at cellular level.

The aforementioned laccase-producing bacterial isolates were selected and characterized for microscopic features. Those four laccase producing isolates (DMC2, DMC3, DMW1, and DMG3) were characterized for Gram reaction features and all found to be purple in color, indicating that they are gram positive as shown in Fig 3. Regarding shape of isolate, all are short rod shaped as indicated in Table 1 and Fig 3.

**Table 1:** Morphological characterization of four selected bacterial isolates from dye mixer.

Sample sources	S.No	Isolates	Microscopic characteristics			Colony morphology
			Gram’s reaction	Shape	Arrangement of the cells	
From dye mixer	1	DMC2	+	Short Rod	Single	Large, translucent, non-filamentous, raised, mucoid and creamy
	2	DMC3	+	Short Rod	Single	Small, creamy, non-filamentous and raised
	3	DMW1	+	Short Rod	Single	white, raised, non filamentous and translucent
	4	DMG3	+	Short Rod	Single	Large, circular, raised, grey and non filamentous

**Fig 3:**
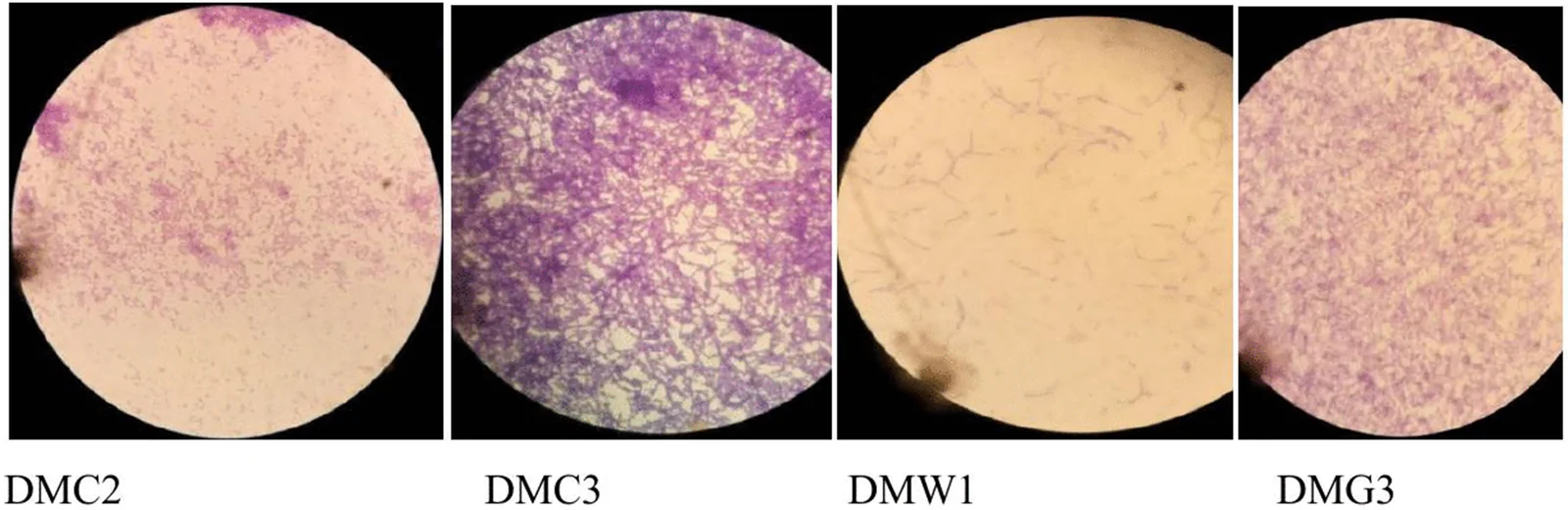
Gram reaction of bacterial isolates.

### 3.5. Optimization of parameters for laccase production

The laccase producing potential of all isolates were checked at different temperature, pH and substrate concentration to optimize the best laccase producing physical and chemical environment (Table 2).

**Table 2:** Biochemical characterization of fourth selected bacterial isolates.

Isolates	Catalase	Urease	Oxidase	Citrate	Indole production	MR	VP	Motility test	TSI				
									Fermentation of sugars			Formation of co_2_	H_2_S Production Test
									Glucose	sucrose	lactose		
DMC2	+	+	+	+	–	–	–	+	+	+	+	+	–
DMC3	+	+	+	+	–	–	–	+	+	+	+	+	–
DMW1	+	+	+	+	–	–	–	+	+	+	+	+	–
DMG3	+	+	+	+	+	–	–	+	+	+	+	+	–

**Effect of pH:** The bacterial isolates were grown on laccase production broth supplemented with dyes and adjusted to varied pH values (ranging from 5 to 8) using buffer. Laccase production was highest at pH7, as indicated in Fig 4. The results showed that DMC2, DMC3, and DMW1 produced high amounts of laccase of 0.643 gm/ml, 0.506 gm/ml, and 0.483 gm/ml, respectively, whereas DMG3 (0.302 gm/mL) produced lowest amounts of laccase.as shown (Fig 4)

**Fig 4:**
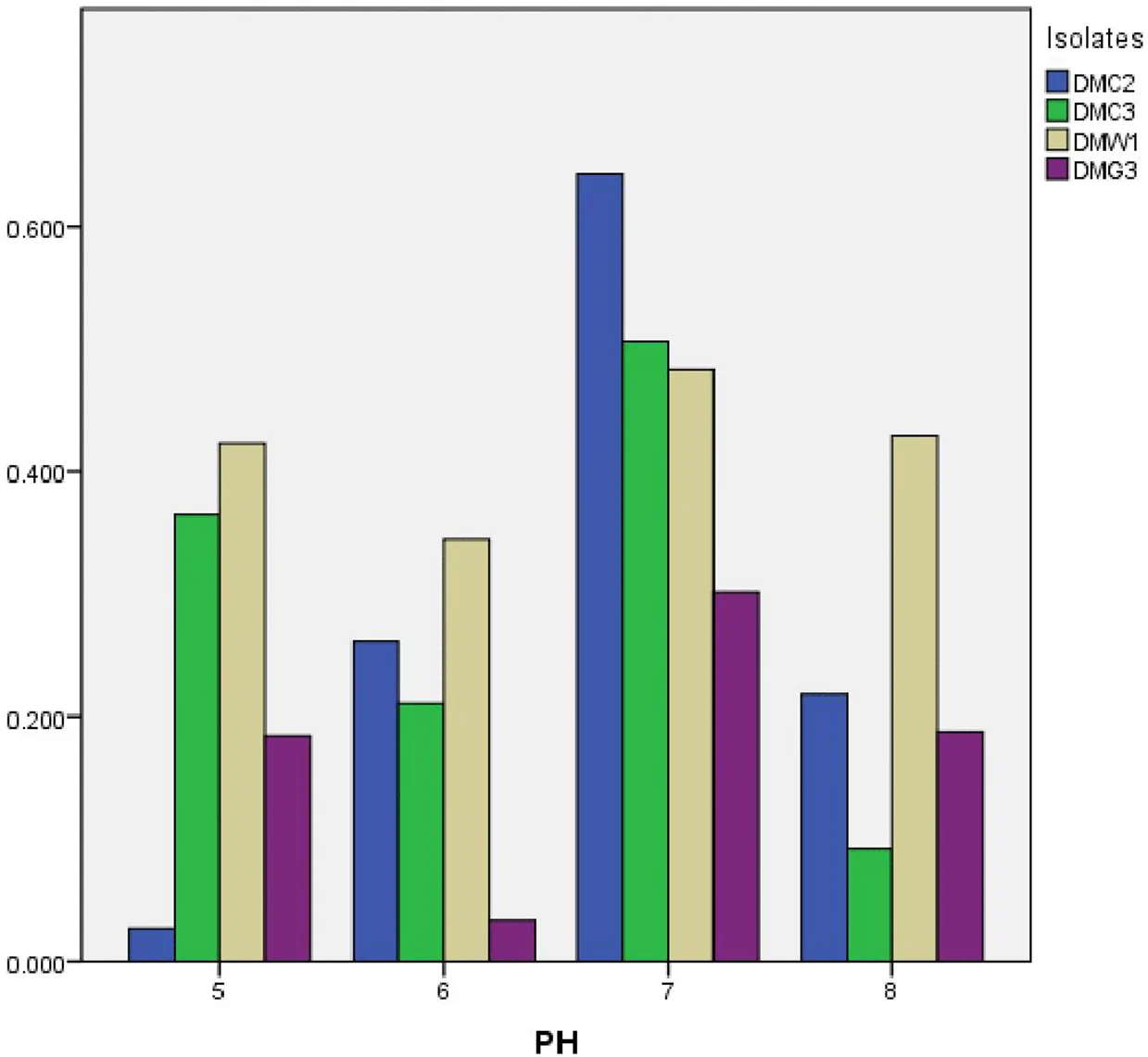
Effect of different levels of pH on laccase production by bacterial isolates.

**Effect of temperatures on laccase production:** The effect of temperature was investigated by incubating the culture at various temperatures (32, 37, 42, and 47∘C). Laccase production was shown to be highest at temperature of 42∘C. The results showed that DMC2, DMC3, and DMW1 produced higher amounts of laccase at 42∘C of 0.643 gm/ml, 0.506 gm/ml, and 0.483 gm/ml, respectively whereas DMG3 produced lowest amount of laccase (0.302 gm/mL) (Fig 5)

**Fig 5:**
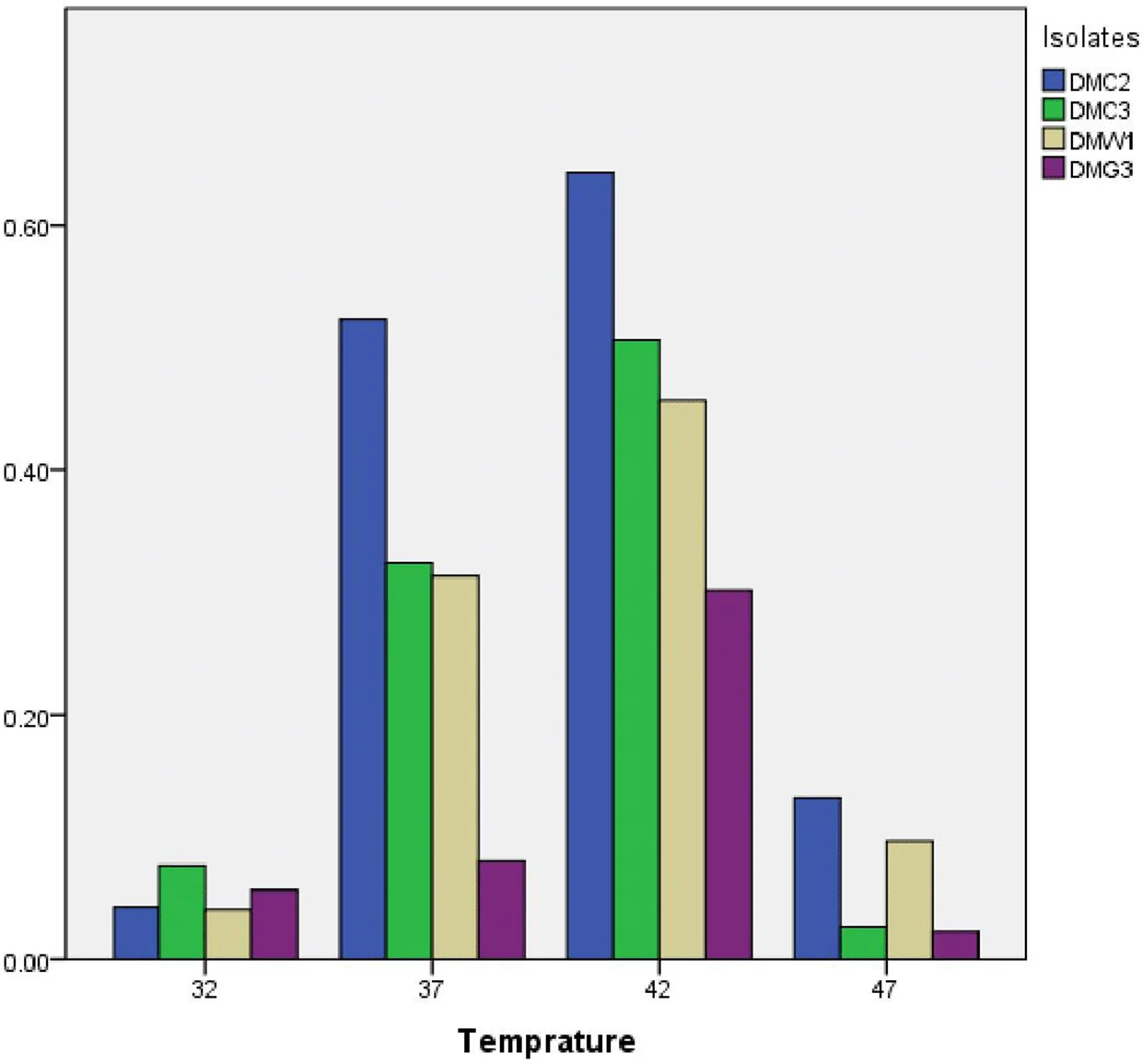
Effect of different temperature on laccase production by bacterial isolates.

**Effect of dye substrate concentrations:** The best concentration of dyes (black, yellow and red) for maximum laccase production were determined by using laccase producing media (LPM) supplemented with different concentrations of dye (0.05 gm, 0.1 gm, and 0.15 gm) (yellow, black, red) (Table 3). Laccase production was found maximum at 0.05 gm of dye substrate as shown in Fig 6. The results showed that DMC2, DMC3 and DMW1 produced maximum amount of laccase, 0.643 gm/ml, 0.506 gm/ml and 0.483 gm/ml respectively whereas DMG3 produced lowest amount of laccase, 0.302 gm/ml at 0.05 gm of dye substrate as shown in (Fig 6).

**Table 3:** Initial and observed absorbance of the three dyes.

Isolates	Initial absorbance of the three selected dyes at appropriate wavelength of each dye			Observed absorbance of the three selected dyes at appropriate wavelength of each dye		
	Black (400nm)	Yellow (580n)	Red (650nm)	Black (400nm)	Yellow (580nm)	Red (650nm)
DMC2	10.822	9.713	7.97	Mean and std. deviation		
				0.015 ± 0.014 mg/ml	0.043 ± 0.031 mg/ml	0.178 ± 0.076 mg/ml
DMC3	10.822	9.713	7.97	0.050 ± 0.009 mg/ml	0.247 ± 0.065 mg/ml	0.293 ± 0.01 mg/ml
DMW1	10.822	9.713	7.97	0.084 ± 0.012 mg/ml	0.119 ± 0.071 mg/ml	0.304 ± 0.149 mg/ml
DMG3	10.822	9.713	7.97	0.107 ± 0.035 mg/ml	0.307 ± 0.022 gm/ml	0.391 ± 0.025 gm/ml

**Fig 6:**
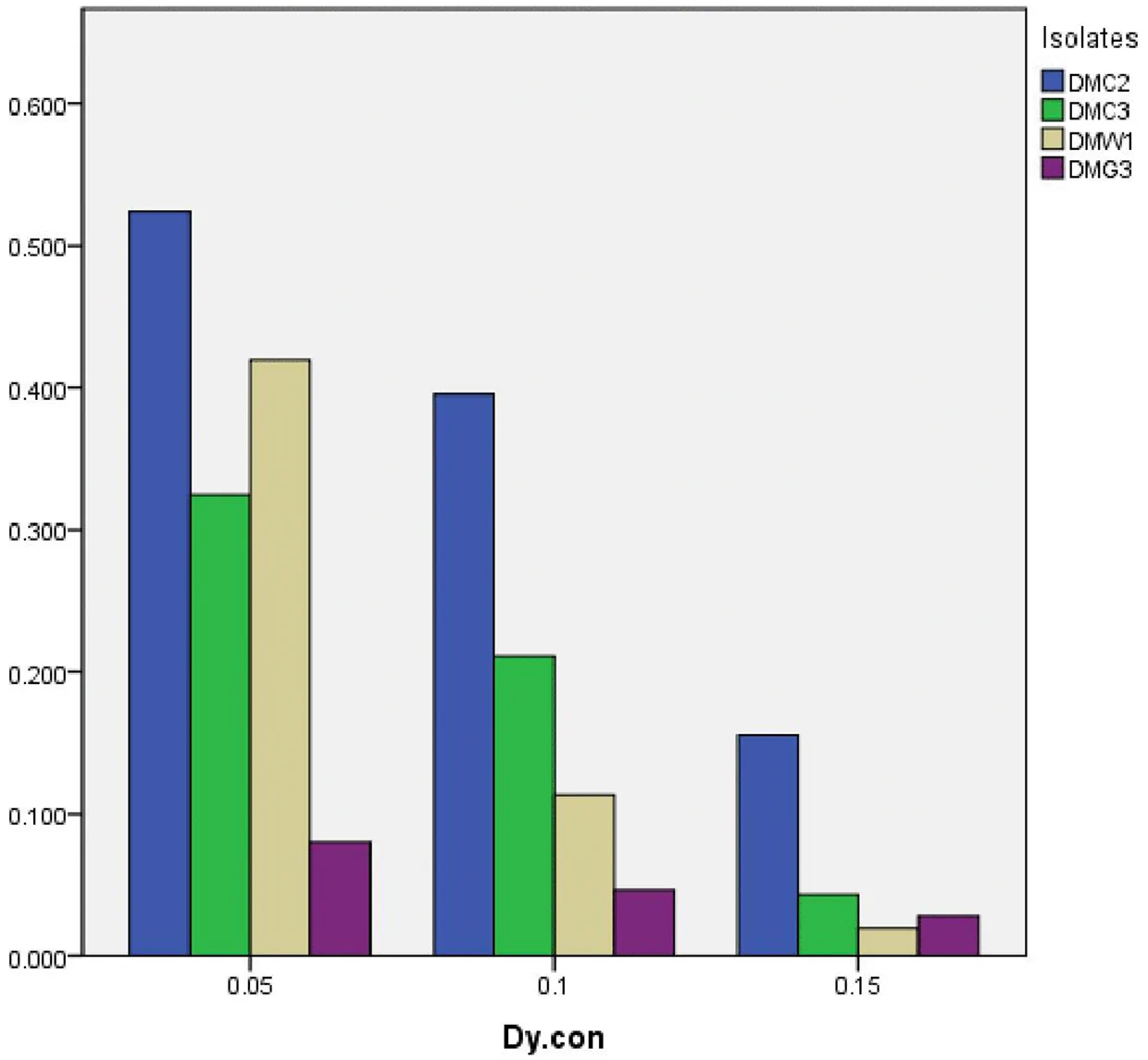
Effect of different dye concentration on laccase production.

### 3.6. Dye decolorization percentage assay by crude laccase enzyme extract

Crude laccase enzymes produced by bacterial isolates (DMC2, DMC3, DMW1, DMG3) were assayed for decolorization efficiency and the results were showed in Table 4 and visualized in Figs 7 and 8. Results of dye decolorization suggest that laccase enzyme of all isolates able to decolorize three dyes (black, yellow and red).

**Table 4:** Decolorization percentage of each dye by using crude laccase enzyme preparation.

The three selected dyes	Decolorization percentage after 48hr			
	Crude laccase enzyme of each isolate			
	Laccase of DMC2	Laccase of DMC3	Laccase of DMW1	Laccase of DMG3
Black	99.861	99.538	99.224	99.011
Yellow	99.557	97.457	98.775	96.839
Red	97.767	96.324	96.186	95.094

**Fig 7:**
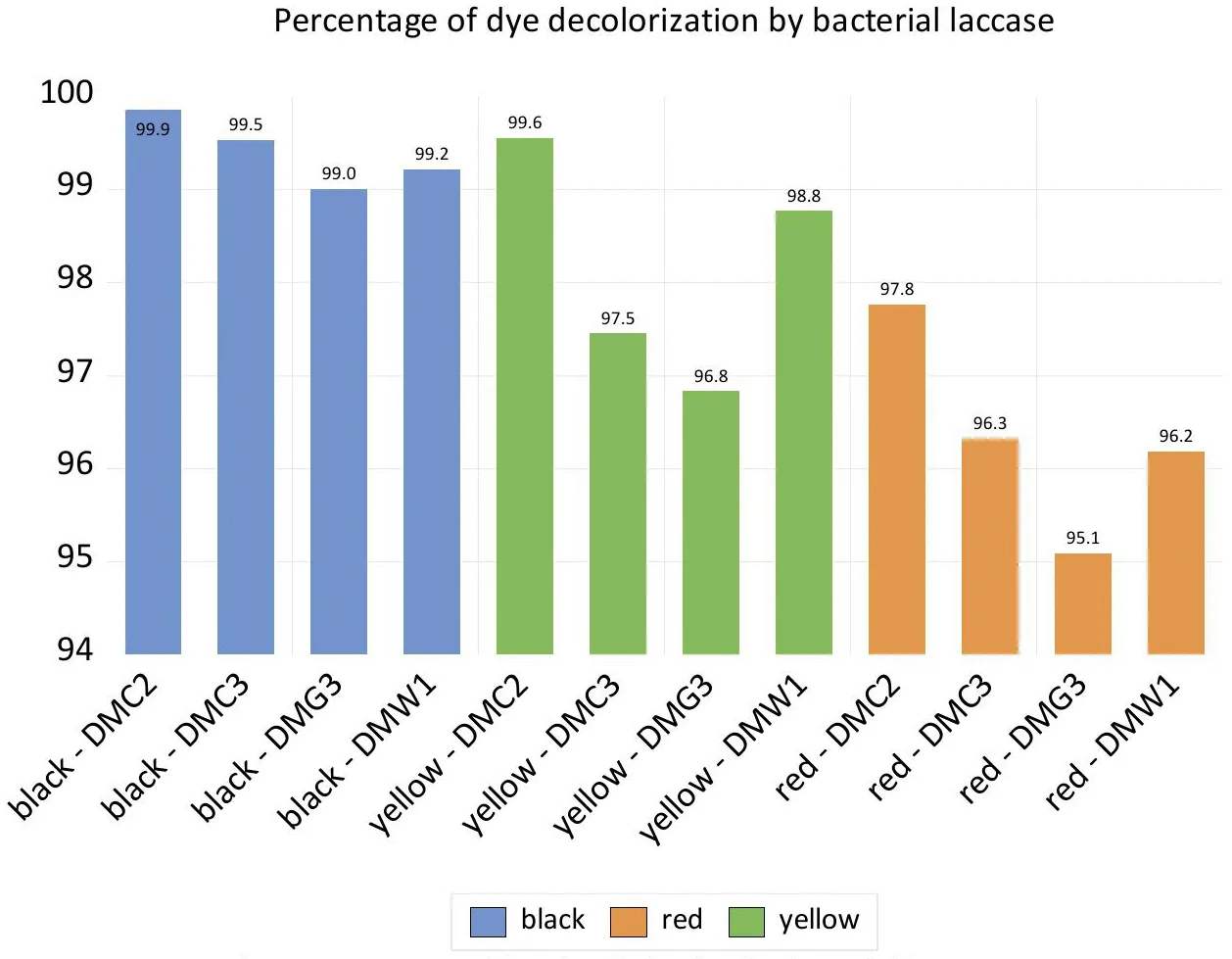
Percentage of dye decolorization by bacterial laccase.

**Fig 8:**
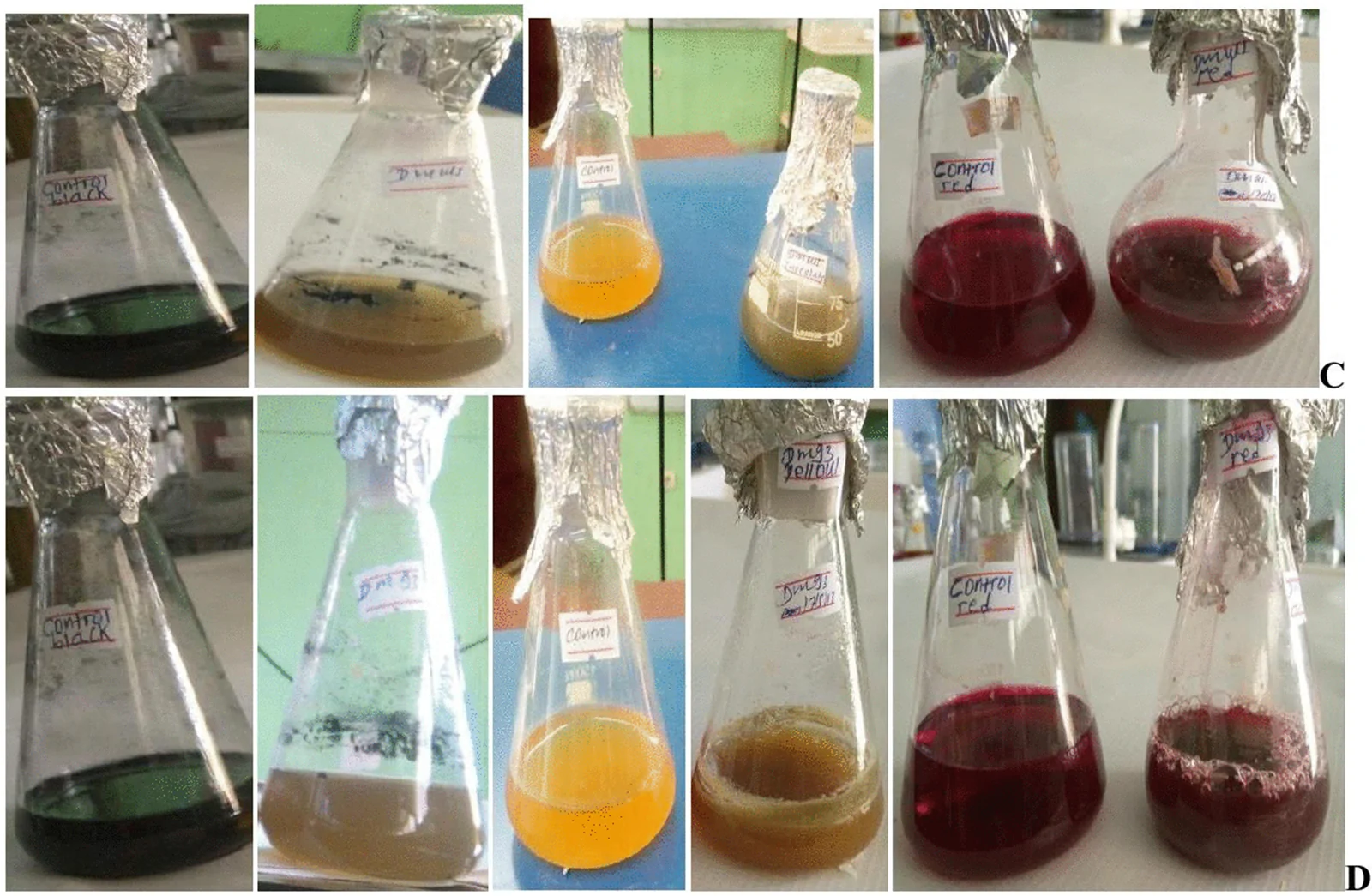
Decolorization of the three dyes by laccase of the isolates.

Decolorization percentage of laccase produced from DMC2, DMC3, DMW1 and DMG3 were increased on black dye (99.861%, 99.538%, 99.224%, and 99.011%, respectively) as compared to the other dyes. Among the laccase enzyme of 4 isolates, laccase enzyme of DMC2 showed higher decolorization efficiency of all dyes for black, yellow, and red dyes with 99.861%, 99.557%, and 97.767%, respectively. This is followed by DMC3, which able to decolorize black, yellow and red dyes with 99.538%, 97.457% and 96.324%, respectively and laccase of DMW1 able to decolorize black, yellow and red dyes with 99.224%, 98.775% and 96.186%, respectively. In addition DMG3 able to decolorize black, yellow and red dyes with 99.011%, 96.839% and 95.094%, respectively.as shown (Fig 8)

## 4. Discussion

In this study, a total of ten bacterial isolates were isolated from the four dyes contaminated wastewater sampling sites, i.e., dye mixer, dye center, dye printing, and dye drain. Six, one, two, and one bacterial isolates were isolated from dye mixer, dye drain, dye center, and dye printing samples, respectively. All isolates were primarily screened using nutrient agar containing 0.5% of tannic acid and they showed black zone surrounding the colonies, which implies tannic acid oxidation. Similarly, [27],used this media for screening of laccase producing bacterial and the isolates were identified as *Bacillus* species. After secondary screening, out of ten, only four isolates, DMC2, DMC3, DMW1 and DMG3 were selected on the basis of laccase production through the formation of the clear zone around the growth of isolates on MSM solid medium containing dye substrates. Thus these isolates were able to degrade dye substrates (black, yellow and red). In connection with this study [17],found that MSM containing dye substrates was used for screening bacterial isolates and the isolates were degrade dyes. This illustrates that the bacterial isolates can synthesize laccase enzyme.

In the present study, four isolates were characterized with phenotypic approach to confirm it at least at genus level. Thus, Bergey’s manual of Systemetic Bacteriology [28],were used to identify the isolates and were belongs to presumptive *Bacillus* genera. A study conducted [29],agreed this result that they were identified as laccase producing *Bacillus* species from dye contaminated wastewater samples in textile industries. Similarly [30],isolated *Bacillus* species from textile dye contaminated wastewater for high laccase production. In addition [31],reported that bacterial isolates were characterized and identified as *Bacillus* species*.*

Physicochemical conditions (temperature, pH and dye concentration) were optimized to pinpoint the suiting conditions for laccase production by the isolates. Statistical analysis exhibited that three bacterial isolates DMC2, DMC3 and DMW1 were statistically significant for laccase production, with values of P’ (0.000 < 0.05) while DMG3 was not statistically significant for the production of laccase with value of P’ (0.270 > 0.05). This indicates that isolates DMC2, DMC3 and DMW1 were a potential laccase enzyme producer with value of 0.643, 0.506 and 0.483 gm/ml, respectively, while isolate DMG3 was lowest laccase producer (0.302 gm/ml).

The maximum laccase production for isolate DMC2, DMC3, DMW1 and DMG3 were 0.643 mg/ml, 0.506 mg/ml, 0.483 mg/ml and 0.302 mg/ml respectively at 42∘C. In other studies, it was noted that, maximum laccase enzyme activity from *Bacillus* species was achieved in the range of 40–50∘C [32]. This is because *Bacillus* species produces high extracellular laccase around 40∘C that is quite near to the present finding [33]. The study of [34], were in accordance with the present result that laccase from *Bacillus* species exhibited maximum activity at 40∘C. Low production of laccase from DMC2, DMG3 and DMW1 were 0.003 mg/ml, 0.009 mg/ml, and 0.004 mg/ml, respectively at 47∘C, whereas low laccase production from DMC3 (0.01 mg/ml) was produced at 32∘C. Increase or decrease the temperature above or below 42∘C, lead to reduce in enzyme production for all the four bacterial isolates.

The higher laccase production for DMC2, DMC3, DMW1 and DMG3 isolates were 0.643 mg/ml, 0.506 mg/ml, 0.483 mg/ml and 0.302 mg/ml, respectively at pH 7. Thus pH in the neutral value was most suitable for laccase production by these all four isolates. Similar study was done by [35], where the optimum pH for laccase production from *Bacillus* species was 7. Moreover [36] found that maximum laccase from *Bacillus* species was produced at pH 7. Low production of laccase from DMC3 (0.01 mg/ml) and DMG3 (0.009 mg/ml) was obtained at PH 6. Low production of laccase from DMW1 (0.004 mg/ml) was obtained at PH 8 whereas low production of laccase from DMC2 (0.003 mg/ml) was produced at PH 5. Similar study was reported by [37], that the optimum pH for maximum laccase production was neutral and the laccase production was decreased rapidly at acid or alkaline pH values. In the present study, increase or decrease pH value above or below 7, lead to reduce in laccase enzyme production.

Maximum production of laccase from DMC2, DMC3, DMW1 and DMG3 was obtained at 0.05 gm of dye concentration with the production of 0.643 mg/ml, 0.506 mg/ml, 0.483 mg/ml and 0.302 mg/ml, respectively. Low production of laccase from DMC2 (0.003 mg/ml), DMG3 (0.009 mg/ml) and DMW1 (0.004 mg/ml) was obtained at 0.15 gm dye concentration whereas low production of laccase from DMC3 (0.023 mg/ml) was produced at 0.1 gm mixed dye concentration. This present study clearly showed that, as the dye concentration was increasing beyond 0.05 gm, laccase enzyme production was decreased. This could be because higher concentration of dye is an inhibitor of bacterial isolates. Similar studies have been reported [38], in literatures where a decrease in the laccase enzyme production was observed with increase in initial dye concentration. With subsequent increase in dye concentration toxic effect of dye and its metabolites became dominant, leading to inhibition in laccase production. The decrease in the efficiency of laccase production with increase in concentration of dye can be due to toxic effect of dye and inadequate amount of biomass to uptake this higher concentration of dye [39].

Decolorization activity was expressed in terms of percentage decolorization and was determined by observing the decrease in absorbance at wavelength of black, yellow and red dyes at 400nm, 580nm and 650nm, respectively. In the present study, crude laccase enzyme preparation produced from bacterial isolates were investigated for three different textile dyes, black, yellow and red which are significant contributors to a serious environmental problem as well as a public health concern and significant results were revealed. The synthetic dye black was decolorized up to 99.861%, 99.538%, 99.224% and 99.011% when treated with a crude laccase enzyme of DMC2, DMC3, DMW1 and DMG3, respectively.

In the case of yellow dye, up to 99.557%, 97.457%, 98.775% and 96.839% when treated with a crude laccase enzyme produced from DMC2, DMC3, DMW1 and DMG3 respectively whereas lesser decolourization was observed with red, 97.767%, 96.324%, 96.186%, and 95.094% when treated with a crude laccase enzyme of DMC2, DMC3, DMW1 and DMG3, respectively within 48hrs of incubation (Table 4). In various studies [40] reported that the *bacillus species*-based laccase enzyme to have a high potential to decolorize/degrade dye which is very similar to this present findings. Similar study has been reported by [41],that decolorization of black dye was found about (96%) by laccase of *Bacillus* species [42] also reported that a maximum decolorization approximately 95 and 85% was recorded for laccase of *Bacillus* species when yellow and red was used, respectively. The variation in the decolorization of the previous studies and the present study might be type of the material they used. The crude laccase enzyme of DMC2 showed greater decolorization percentages for all dyes, compared to the crude laccase enzyme of DMC3, DMW1 and DMG3. The difference in decolorization pattern is due to the structure and complexity and the interaction with azo bond with different dyes as reported by [43]

## 5. Conclussion

In the present study, an extracellular laccase producing bacteria was isolated and identified as *Bacillus* species. maximum laccase production was obtained at 0.05 g dye concentration, together with the optimum conditions of pH 7 and 42°C, demonstrating the suitability of the isolated Bacillus species for textile dye bioremediation applications. Under these optimized conditions, isolate DMC2 produced the highest laccase concentration (0.643 mg/mL) and achieved dye decolorization efficiencies of 99.861%, 99.557%, and 97.767% for black, yellow, and red dyes, respectively. So, this investigation concluded *Bacillus* species has enormous potentials in the bioremediation of textile dye wastewater. Therefore, the results obtained make laccase from this microorganism potentially useful target in several biotechnological applications such as in bio-catalysis and have a possible role in bioremediation of textile dye wastewater. Difference decolorization pattern was observed among the dyes. As a result, the crude laccase enzyme from these bacterial isolates might be employed since a viable alternative to the physical and chemical processes of textile dye, as they have a high potential for decolorizing or degrading the dyes in this study.

## 6. Recommendation

Based on the findings of this study the following points are recommended

- Scale up research is important for laccase enzyme synthesis in the dye decolorization process to a specified industrial level.
- Extracellular laccase enzyme should be purified wherever possible.
- Other toxic chemicals that arise from the textile factory should be treated
- Furthermore, molecular identification is required to correctly identify laccase producing isolates up to species and strain level.

## Supporting information

S1 File (DOCX)

S2 File (DOCX)
