# Supplementary material for: Isolation, characterization, and optimization of extracellular laccase-producing bacteria from dye-contaminated textile wastewater for dye decolonization
Source: PLoS One. 2026 Sep 29;21(9):e0354516. doi: 10.1371/journal.pone.0354516 (PMC13623100; doi:10.1371/journal.pone.0354516)
Supplement: Supplementary file 1 [file pone.0354516.s001.docx]

Appendix

Catalase Test	Oxidase Test

A

Slant Urease Test	Slant citrate test	Slant TSI test	Motility test

Fig9. Some selected pictures: the pictures represent important laboratory works: (A) indicates the biochemical test: urease, Citrate, TSI and motility biochemical test results respectively.
