# Supplementary material for: Isolation, characterization, and optimization of extracellular laccase-producing bacteria from dye-contaminated textile wastewater for dye decolonization
Source: PLoS One. 2026 Sep 29;21(9):e0354516. doi: 10.1371/journal.pone.0354516 (PMC13623100; doi:10.1371/journal.pone.0354516)
Supplement: Supplementary file 2 [file pone.0354516.s002.docx]

Table 5: Descriptive statistics analysis

| Isolates | Mean | Standard deviation | Sum of square | Mean square | P |
| --- | --- | --- | --- | --- | --- |
| DMC2 | 0.182 | 0.191 | 0.815 | 0.272 | 0.00^a^ |
| DMC3 | 0.135 | 0.156 | 0.603 | 0.201 | 0.00^a^ |
| DMW1 | 0.165 | 0.165 | 0.507 | 0.169 | 0.00^a^ |
| DMG3 | 0.074 | 0.083 | 0.027 | 0.009 | 0.270^b^ |

|  | Color |  | | |  | | |  | | |
| --- | --- | --- | --- | --- | --- | --- | --- | --- | --- | --- |
| S.no | Isolate | Yellow | | | Red | | | Black | |  |
| 1 | DMC2 | 99.53 | 99.67 | 99.60 | 97.71 | 97.80 | 97.89 | 99.9 | 99.9 | 99.9 |
| 2 | DMC3 | 97.42 | 97.58 | 97.50 | 96.22 | 96.30 | 96.38 | 99.4 | 99.6 | 99.5 |
| 3 | DMG3 | 96.72 | 96.81 | 96.87 | 95.02 | 95.10 | 95.18 | 99.1 | 98.7 | 99.2 |
| 4 | DMW1 | 98.72 | 98.80 | 98.88 | 96.11 | 96.20 | 96.29 | 99 | 99.2 | 99.4 |
|  | R=replicate | R1 | R2 | R3 | R1 | R2 | R3 | R1 | R2 | R3 |

Raw date for Percentage of dye decolorization by bacterial laccase

Descriptive statistics analysis raw data

| Isolates | R1 | R2 | R3 |
| --- | --- | --- | --- |
| DMC2 | 0.071 | 0.187 | 0.288 |
| DMC3 | 0.029 | 0.135 | 0.241 |
| DMW1 | 0.044 | 0.165 | 0.286 |
| DMG3 | 0.017 | 0.074 | 0.131 |
