# Supplementary material for: Isolation, characterization, and optimization of extracellular laccase-producing bacteria from dye-contaminated textile wastewater for dye decolonization
Source: PLoS One. 2026 Sep 29;21(9):e0354516. doi: 10.1371/journal.pone.0354516 (PMC13623100; doi:10.1371/journal.pone.0354516)
Supplement: Supplementary file 3 [file pone.0354516.s003.docx]

Reviewer 1 comment response

|  | Old one | Rivesed one |
| --- | --- | --- |
| Tittle | Isolation and Characterization of Extracellular Laccase Producing Bacteria from Dye-Contaminated Textile Wastewater for Dye Decolorization | **Isolation, Characterization, and Optimization of Extracellular Laccase-Producing Bacteria from Dye-Contaminated Textile Wastewater for Dye Decolonization** |
| Abstract |  | Maximum laccase production and dye decolorization were achieved at pH 7, a temperature of 42°C, and a dye concentration of 0.05 g per 50 mL of culture medium. |
| Introduction |  | The Introduction has been expanded to include other importantdye-degrading enzymes such as lignin peroxidase(LiP),manganese peroxidase(MnP),azoreductase, tyrosinase, and versatile peroxidase  And the objective is corrected as  Therefore, this study was to isolate, screen and characterize laccase-producing bacteria from textile dye-contaminated wastewater Textile Factory for the purpose of decolorizing Textile dye |
| Methods | Effect of Temperature  Effect of pH  Effect of Dye Concentration | 3.9.1 Effect of Temperature  3.9.2 Effect of pH  3.9.3 Effect of Dye Concentration |
|  |  | 3.3.1 **Reagents and Chemicals**  The reagents and chemicals used in the study included nutrient agar components (peptone, beef extract, sodium chloride, and agar), physiological saline water, tannic acid, glucose, yeast extract, calcium c arbonate (CaCO₃), copper sulfate (CuSO₄), magnesium sulfate (MgSO₄), ferrous sulfate (FeSO₄), zinc sulfate (ZnSO₄), and textile dyes (yellow, black, and red). Various biochemical test reagents were also employed, including hydrogen peroxide (3% H₂O₂), urea agar, Triple Sugar Iron (TSI) agar, MR-VP broth, methyl red indicator, α-naphthol, potassium hydroxide (KOH), Simmons citrate agar, tryptophan broth, and Kovac’s reagent. Additional media components used for laccase production included tryptone and buffer solutions for pH adjustment. These chemicals and reagents were essential for bacterial isolation, screening, biochemical characterization, optimization of laccase production, and evaluation of dye degradation potential |
| Table |  | All tables and figures have been reformatted according to the journal's author guidelines, including caption style, numbering, alignment, and presentation format |
| conclusion |  | The conclusion has been revised to include the optimum dye concentration results. The revised statement indicates that maximum laccase production was obtained at **0.05 g dye concentration**, together with the optimum conditions of **pH 7** and **42°C**, demonstrating the suitability of the isolated Bacillus species for textile dye bioremediation applications |

Reviewer 2 comment response

| Introduction |  |  |
| --- | --- | --- |
|  | The introduction and results and discussion sections can be supported by citing recent references that study the treatment of textile effluents | We have carefully reviewed the recommended publications and incorporated relevant citations throughout the Introduction, Results, and Discussion sections. The newly added references strengthen the background information on textile wastewater pollution, recent advances in biological treatment technologies, enzyme-mediated dye degradation, and sustainable bioremediation approaches. These references also provide additional support for our findings on bacterial laccase-mediated dye decolorization and optimization of treatment conditions. |
|  | objective | Therefore, this study was to isolate, screen and characterize laccase-producing bacteria from textile dye-contaminated wastewater Textile Factory for the purpose of decolorizing Textile dye |
|  | Typos and grammatical errors must be corrected throughout the manuscript | The entire manuscript has been thoroughly revised for grammar, spelling, sentence structure, punctuation, consistency of scientific terminology, and formatting |
| Conclusion |  | The maximum laccase production was obtained at **0.05 g dye concentration**, together with the optimum conditions of **pH 7** and **42°C**, demonstrating the suitability of the isolated Bacillus species for textile dye bioremediation applications. Under these optimized conditions, isolate DMC2 produced the highest laccase concentration (0.643 mg/mL) and achieved dye decolorization efficiencies of 99.861%, 99.557%, and 97.767% for black, yellow, and red dyes, respectively. |
| Formatting |  | The numbering format of all manuscript sections and subsections has been carefully reviewed and revised to ensure consistency with the journal guidelines. Section headings, subsection numbering, figure numbering, table numbering, and equation numbering have been corrected and standardized throughout the manuscript |
