# Supplementary material for: Isolation, characterization, and optimization of extracellular laccase-producing bacteria from dye-contaminated textile wastewater for dye decolonization
Source: PLoS One. 2026 Sep 29;21(9):e0354516. doi: 10.1371/journal.pone.0354516 (PMC13623100; doi:10.1371/journal.pone.0354516)
Supplement: Peer review history

# Peer review: Decision Letter 0

**DOI:** 10.1371/journal.pone.0354516.r001

**Authors:** Habibullah Nadeem

13 May 2026

Dear Dr. getachew,

Thank you for submitting your manuscript to PLOS ONE. After careful consideration, we feel that it has merit but does not fully meet PLOS ONE’s publication criteria as it currently stands. Therefore, we invite you to submit a revised version of the manuscript that addresses the points raised during the review process.

Please submit your revised manuscript by Jun 27 2026 11:59PM. If you will need more time than this to complete your revisions, please reply to this message or contact the journal office at plosone@plos.org. When you're ready to submit your revision, log on to https://www.editorialmanager.com/pone/ and select the 'Submissions Needing Revision' folder to locate your manuscript file.

- A letter that responds to each point raised by the academic editor and reviewer(s). You should upload this letter as a separate file labeled 'Response to Reviewers'.
- A marked-up copy of your manuscript that highlights changes made to the original version. You should upload this as a separate file labeled 'Revised Manuscript with Track Changes'.
- An unmarked version of your revised paper without tracked changes. You should upload this as a separate file labeled 'Manuscript'.

If you would like to make changes to your financial disclosure, please include your updated statement in your cover letter. Guidelines for resubmitting your figure files are available below the reviewer comments at the end of this letter.

If applicable, we recommend that you deposit your laboratory protocols in protocols.io to enhance the reproducibility of your results. Protocols.io assigns your protocol its own identifier (DOI) so that it can be cited independently in the future. For instructions see: https://journals.plos.org/plosone/s/submission-guidelines#loc-laboratory-protocols. Additionally, PLOS ONE offers an option for publishing peer-reviewed Lab Protocol articles, which describe protocols hosted on protocols.io. Read more information on sharing protocols at https://plos.org/protocols?utm_medium=editorial-email&utm_source=authorletters&utm_campaign=protocols.

As the corresponding author, your ORCID iD is verified in the submission system and will appear in the published article. PLOS supports the use of ORCID, and we encourage all coauthors to register for an ORCID iD and use it as well. Please encourage your coauthors to verify their ORCID iD within the submission system before final acceptance, as unverified ORCID iDs will not appear in the published article. *Only*  the individual author can complete the verification step; PLOS staff *cannot*  verify ORCID iDs on behalf of authors.

We look forward to receiving your revised manuscript.

Kind regards,

Habibullah Nadeem

Academic Editor

PLOS One

**Journal Requirements:**

When submitting your revision, we need you to address these additional requirements.

1. Please ensure that your manuscript meets PLOS ONE's style requirements, including those for file naming. The PLOS ONE style templates can be found at

https://journals.plos.org/plosone/s/file?id=wjVg/PLOSOne_formatting_sample_main_body.pdf and

https://journals.plos.org/plosone/s/file?id=ba62/PLOSOne_formatting_sample_title_authors_affiliations.pdf

2. Thank you for stating the following in your Competing Interests section:

“no competing interest.”

Please complete your Competing Interests on the online submission form to state any Competing Interests. If you have no competing interests, please state "The authors have declared that no competing interests exist.", as detailed online in our guide for authors at http://journals.plos.org/plosone/s/submit-now

This information should be included in your cover letter; we will change the online submission form on your behalf.

3. We note that you have indicated that there are restrictions to data sharing for this study. PLOS only allows data to be available upon request if there are legal or ethical restrictions on sharing data publicly. For more information on unacceptable data access restrictions, please see http://journals.plos.org/plosone/s/data-availability#loc-unacceptable-data-access-restrictions.

Before we proceed with your manuscript, please address the following prompts:

a) If there are ethical or legal restrictions on sharing a de-identified data set, please explain them in detail (e.g., data contain potentially identifying or sensitive patient information, data are owned by a third-party organization, etc.) and who has imposed them (e.g., a Research Ethics Committee or Institutional Review Board, etc.). Please also provide contact information for a data access committee, ethics committee, or other institutional body to which data requests may be sent.

b) If there are no restrictions, please upload the minimal anonymized data set necessary to replicate your study findings to a stable, public repository and provide us with the relevant URLs, DOIs, or accession numbers. For a list of recommended repositories, please see

https://journals.plos.org/plosone/s/recommended-repositories. You also have the option of uploading the data as Supporting Information files, but we would recommend depositing data directly to a data repository if possible.

We will update your Data Availability statement on your behalf to reflect the information you provide.

4. PLOS requires an ORCID iD for the corresponding author in Editorial Manager on papers submitted after December 6th, 2016. Please ensure that you have an ORCID iD and that it is validated in Editorial Manager. To do this, go to ‘Update my Information’ (in the upper left-hand corner of the main menu), and click on the Fetch/Validate link next to the ORCID field. This will take you to the ORCID site and allow you to create a new iD or authenticate a pre-existing iD in Editorial Manager.

5. Please upload a copy of Figures 5, 6, 7 and 8 to which you refer in your text on pages 16, 17, and 18. If the figure is no longer to be included as part of the submission please remove all reference to it within the text.

6. We note that Figures in file (Figure 1.docx) in your submission contain copyrighted images. All PLOS content is published under the Creative Commons Attribution License (CC BY 4.0), which means that the manuscript, images, and Supporting Information files will be freely available online, and any third party is permitted to access, download, copy, distribute, and use these materials in any way, even commercially, with proper attribution. For more information, see our copyright guidelines: http://journals.plos.org/plosone/s/licenses-and-copyright.

We require you to either (1) present written permission from the copyright holder to publish these figures specifically under the CC BY 4.0 license, or (2) remove the figures from your submission:

1. You may seek permission from the original copyright holder Figures in file (Figure 1.docx)  to publish the content specifically under the CC BY 4.0 license.

We recommend that you contact the original copyright holder with the Content Permission Form (http://journals.plos.org/plosone/s/file?id=7c09/content-permission-form.pdf) and the following text:

“I request permission for the open-access journal PLOS ONE to publish XXX under the Creative Commons Attribution License (CCAL) CC BY 4.0 (http://creativecommons.org/licenses/by/4.0/). Please be aware that this license allows unrestricted use and distribution, even commercially, by third parties. Please reply and provide explicit written permission to publish XXX under a CC BY license and complete the attached form.”

Please upload the completed Content Permission Form or other proof of granted permissions as an "Other" file with your submission.

In the figure caption of the copyrighted figure, please include the following text: “Reprinted from [ref] under a CC BY license, with permission from [name of publisher], original copyright [original copyright year].”

2. If you are unable to obtain permission from the original copyright holder to publish these figures under the CC BY 4.0 license or if the copyright holder’s requirements are incompatible with the CC BY 4.0 license, please either i) remove the figure or ii) supply a replacement figure that complies with the CC BY 4.0 license. Please check copyright information on all replacement figures and update the figure caption with source information. If applicable, please specify in the figure caption text when a figure is similar but not identical to the original image and is therefore for illustrative purposes only.

7. We note you have included a table to which you do not refer in the text of your manuscript. Please ensure that you refer to Tables 2 and 3 in your text; if accepted, production will need this reference to link the reader to the Table.

8. Please include captions for your Supporting Information files at the end of your manuscript, and update any in-text citations to match accordingly. Please see our Supporting Information guidelines for more information: http://journals.plos.org/plosone/s/supporting-information.

9. If the reviewer comments include a recommendation to cite specific previously published works, please review and evaluate these publications to determine whether they are relevant and should be cited. There is no requirement to cite these works unless the editor has indicated otherwise.

10. Please review your reference list to ensure that it is complete and correct. If you have cited papers that have been retracted, please include the rationale for doing so in the manuscript text, or remove these references and replace them with relevant current references. Any changes to the reference list should be mentioned in the rebuttal letter that accompanies your revised manuscript. If you need to cite a retracted article, indicate the article’s retracted status in the References list and also include a citation and full reference for the retraction notice.

[Note: HTML markup is below. Please do not edit.]

Reviewers' comments:

Reviewer's Responses to Questions

**Comments to the Author**

1. Is the manuscript technically sound, and do the data support the conclusions?

Reviewer #1: Yes

Reviewer #2: Partly

**********

2. Has the statistical analysis been performed appropriately and rigorously?

Reviewer #1: Yes

Reviewer #2: Yes

**********

3. Have the authors made all data underlying the findings in their manuscript fully available?

The PLOS Data policy

Reviewer #1: Yes

Reviewer #2: Yes

**********

4. Is the manuscript presented in an intelligible fashion and written in standard English?

Reviewer #1: Yes

Reviewer #2: Yes

**********

Reviewer #1: 1. The introduction and results and discussion sections can be supported by citing recent references that study the treatment of textile effluents. Citation of the following references will help the authors to boost the readership of this manuscript. The following are some suggestions

https://doi.org/10.55730/1300-0527.3517; https://doi.org/10.1007/s12221-025-01208-4; https://doi.org/10.31881/TLR.2023.165; https://doi.org/10.1108/RJTA-10-2021-0124; https://doi.org/10.1007/s12221-024-00712-3

2. Typos and grammatical errors must be corrected throughout the manuscript.

3. The objective of the work is not clearly mentioned in the end of the introduction part. The novelty of this research must be highlighted under the Introduction section.

4. The conclusion needs to be written with the numerical value obtained with the present research work.

5. The numeration of sections should be revised

Reviewer #2: The research work addressed key issue in environmental pollution, especially water pollution caused by dyes which might be toxic to both aquatic ecosystems. The biological approach using bacteria to extract the needed enzymes for biodegradation is innovative and commendable. This gave the academic merit to the quality of research work. However, the manuscript still requires minor revision before considered for publication. The authors' need to address the below comments to further improve their quality of research works.

Title

1. The title needs to be reframed to aligned with the manuscript's content. I suggest the authors include optimization in the title.

Abstract

1. The authors should specific the optimization variables using appropriate units. pH, temperature, Concentration. The authors should check the statement ...with 0.05gm dye concentration at 42 and p H 7 and reframe for clarity.

Introduction

1. The authors need to provide other biological source of the dye's degradation enzymes apart from the bacteria in the test.

2. The authors provide information of other dye's degradation enzymes apart from laccase.

3. The authors should the main objectives of the study in the last paragraph of the introduction section

Method

1. The authors should provide details information on the reagent and chemicals used in this study. Provide subtitle for this comment.

2. The 3.9. section should be subtitle accordingly. effect of temperature, p H, dye concentration.

3. The % Decolorization calculation should be numbered and refer to in the write up (Page 11)

Results

1. The tables and figures should be presented in line with the manuscript's authors guideline.

2.The error bar notation should the included in the figures provided.

Discussion

1. The authors should remove the subtitles from this section.

2. The authors should provide more similar research work literature in this section to further justify their findings.

Conclusion

1. The authors should findings for concentration optimization.

References

1. The authors need to check the references section for errors in punctuation and uniform referencing's styles.

General

The authors need to format the write up in line with the journal authors guidelines.

**********

what does this mean?). If published, this will include your full peer review and any attached files.

If you choose “no”, your identity will remain anonymous but your review may still be made public.

**Do you want your identity to be public for this peer review?**  For information about this choice, including consent withdrawal, please see our Privacy Policy

Reviewer #1: No

Reviewer #2: **Yes:**  FOLUSO OLUWAGBEMIGA OSUNSANMI

**********

[NOTE: If reviewer comments were submitted as an attachment file, they will be attached to this email and accessible via the submission site. Please log into your account, locate the manuscript record, and check for the action link "View Attachments". If this link does not appear, there are no attachment files.]

To ensure your figures meet our technical requirements, please review our figure guidelines: https://journals.plos.org/plosone/s/figures

You may also use PLOS’s free figure tool, NAAS, to help you prepare publication quality figures: https://journals.plos.org/plosone/s/figures#loc-tools-for-figure-preparation.

NAAS will assess whether your figures meet our technical requirements by comparing each figure against our figure specifications.

# Author comment: Author response to Decision Letter 1

**DOI:** 10.1371/journal.pone.0354516.r002

1 Jul 2026

Reviewer 1 comment response

Old one Rivesed one

Tittle Isolation and Characterization of Extracellular Laccase Producing Bacteria from Dye-Contaminated Textile Wastewater for Dye Decolorization

Isolation, Characterization, and Optimization of Extracellular Laccase-Producing Bacteria from Dye-Contaminated Textile Wastewater for Dye Decolonization

Abstract Maximum laccase production and dye decolorization were achieved at pH 7, a temperature of 42°C, and a dye concentration of 0.05 g per 50 mL of culture medium.

Introduction The Introduction has been expanded to include other importantdye-degrading enzymes such as lignin peroxidase(LiP),manganese peroxidase(MnP),azoreductase, tyrosinase, and versatile peroxidase

And the objective is corrected as

Therefore, this study was to isolate, screen and characterize laccase-producing bacteria from textile dye-contaminated wastewater Textile Factory for the purpose of decolorizing Textile dye

Methods Effect of Temperature

Effect of pH

Effect of Dye Concentration 3.9.1 Effect of Temperature

3.9.2 Effect of pH

3.9.3 Effect of Dye Concentration

3.3.1 Reagents and Chemicals

The reagents and chemicals used in the study included nutrient agar components (peptone, beef extract, sodium chloride, and agar), physiological saline water, tannic acid, glucose, yeast extract, calcium c arbonate (CaCO₃), copper sulfate (CuSO₄), magnesium sulfate (MgSO₄), ferrous sulfate (FeSO₄), zinc sulfate (ZnSO₄), and textile dyes (yellow, black, and red). Various biochemical test reagents were also employed, including hydrogen peroxide (3% H₂O₂), urea agar, Triple Sugar Iron (TSI) agar, MR-VP broth, methyl red indicator, α-naphthol, potassium hydroxide (KOH), Simmons citrate agar, tryptophan broth, and Kovac’s reagent. Additional media components used for laccase production included tryptone and buffer solutions for pH adjustment. These chemicals and reagents were essential for bacterial isolation, screening, biochemical characterization, optimization of laccase production, and evaluation of dye degradation potential

Table All tables and figures have been reformatted according to the journal's author guidelines, including caption style, numbering, alignment, and presentation format

conclusion The conclusion has been revised to include the optimum dye concentration results. The revised statement indicates that maximum laccase production was obtained at 0.05 g dye concentration, together with the optimum conditions of pH 7 and 42°C, demonstrating the suitability of the isolated Bacillus species for textile dye bioremediation applications

Reviewer 2 comment response

Introduction

The introduction and results and discussion sections can be supported by citing recent references that study the treatment of textile effluents We have carefully reviewed the recommended publications and incorporated relevant citations throughout the Introduction, Results, and Discussion sections. The newly added references strengthen the background information on textile wastewater pollution, recent advances in biological treatment technologies, enzyme-mediated dye degradation, and sustainable bioremediation approaches. These references also provide additional support for our findings on bacterial laccase-mediated dye decolorization and optimization of treatment conditions.

objective

Therefore, this study was to isolate, screen and characterize laccase-producing bacteria from textile dye-contaminated wastewater Textile Factory for the purpose of decolorizing Textile dye

Typos and grammatical errors must be corrected throughout the manuscript The entire manuscript has been thoroughly revised for grammar, spelling, sentence structure, punctuation, consistency of scientific terminology, and formatting

Conclusion The maximum laccase production was obtained at 0.05 g dye concentration, together with the optimum conditions of pH 7 and 42°C, demonstrating the suitability of the isolated Bacillus species for textile dye bioremediation applications. Under these optimized conditions, isolate DMC2 produced the highest laccase concentration (0.643 mg/mL) and achieved dye decolorization efficiencies of 99.861%, 99.557%, and 97.767% for black, yellow, and red dyes, respectively.

Formatting The numbering format of all manuscript sections and subsections has been carefully reviewed and revised to ensure consistency with the journal guidelines. Section headings, subsection numbering, figure numbering, table numbering, and equation numbering have been corrected and standardized throughout the manuscript

Attachment Submitted filename: Reviewer 1 and 2comment response.docx

# Editor report: Decision Letter 1

**DOI:** 10.1371/journal.pone.0354516.r003

**Authors:** Habibullah Nadeem

10 Jul 2026

Isolation and Characterization of Extracellular Laccase Producing Bacteria

from Dye-Contaminated Textile Wastewater for Dye Decolorization

PONE-D-26-07255R1

Dear Dr. getachew,

We’re pleased to inform you that your manuscript has been judged scientifically suitable for publication and will be formally accepted for publication once it meets all outstanding technical requirements.

Within one week, you’ll receive an e-mail detailing the required amendments. When these have been addressed, you’ll receive a formal acceptance letter and your manuscript will be scheduled for publication.

An invoice will be generated when your article is formally accepted. Please note, if your institution has a publishing partnership with PLOS and your article meets the relevant criteria, all or part of your publication costs will be covered. Please make sure your user information is up-to-date by logging into Editorial Manager at Editorial Manager® and clicking the ‘Update My Information' link at the top of the page. For questions related to billing, please contact billing support.

If your institution or institutions have a press office, please notify them about your upcoming paper to help maximize its impact. If they’ll be preparing press materials, please inform our press team as soon as possible -- no later than 48 hours after receiving the formal acceptance. Your manuscript will remain under strict press embargo until 2 pm Eastern Time on the date of publication. For more information, please contact onepress@plos.org.

Kind regards,

Habibullah Nadeem

Academic Editor

PLOS One

Additional Editor Comments (optional):

Reviewers' comments:
